# Pressure induced structural phase crossover of a GaSe epilayer grown under screw dislocation driven mode and its phase recovery

**DOI:** 10.1038/s41598-021-99419-1

**Published:** 2021-10-06

**Authors:** Nhu Quynh Diep, Ssu Kuan Wu, Cheng Wei Liu, Sa Hoang Huynh, Wu Ching Chou, Chih Ming Lin, Dong Zhou Zhang, Ching Hwa Ho

**Affiliations:** 1 Department of Electrophysics, College of Sciences, National Yang-Ming Chiao-Tung University, Hsinchu, 30010 Taiwan; 2 Department of Physics, College of Sciences, National Tsing Hua University, Hsinchu, 300044 Taiwan; 3 GeoSoilEnviroCARS, Argonne National Laboratory, 9700 S Cass Ave, Lemont, 60439 IL USA; 4 Graduate Institute of Applied Science and Technology, National Taiwan University of Science and Technology, Taipei, 106 Taiwan

**Keywords:** Two-dimensional materials, Phase transitions and critical phenomena

## Abstract

Hydrostatically pressurized studies using diamond anvil cells on the structural phase transition of the free-standing screw-dislocation-driven (SDD) GaSe thin film synthesized by molecular beam epitaxy have been demonstrated via in-situ angle-dispersive synchrotron X-ray diffraction and Raman spectroscopy. The early pressure-driven hexagonal-to-rock salt transition at approximately ~ 20 GPa as well as the outstandingly structural-phase memory after depressurization in the SDD-GaSe film was recognized, attributed to the screw dislocation-assisted mechanism. Note that, the reversible pressure-induced structural transition was not evidenced from the GaSe bulk, which has a layer-by-layer stacking structure. In addition, a remarkable 1.7 times higher in bulk modulus of the SDD-GaSe film in comparison to bulk counterpart was observed, which was mainly contributed by its four times higher in the incompressibility along *c*-axis. This is well-correlated to the slower shifting slopes of out-of-plane phonon-vibration modes in the SDD-GaSe film, especially at low-pressure range (< 5 GPa). As a final point, we recommend that the intense density of screw dislocation cores in the SDD-GaSe lattice structure plays a crucial role in these novel phenomena.

## Introduction

**Figure 1:**
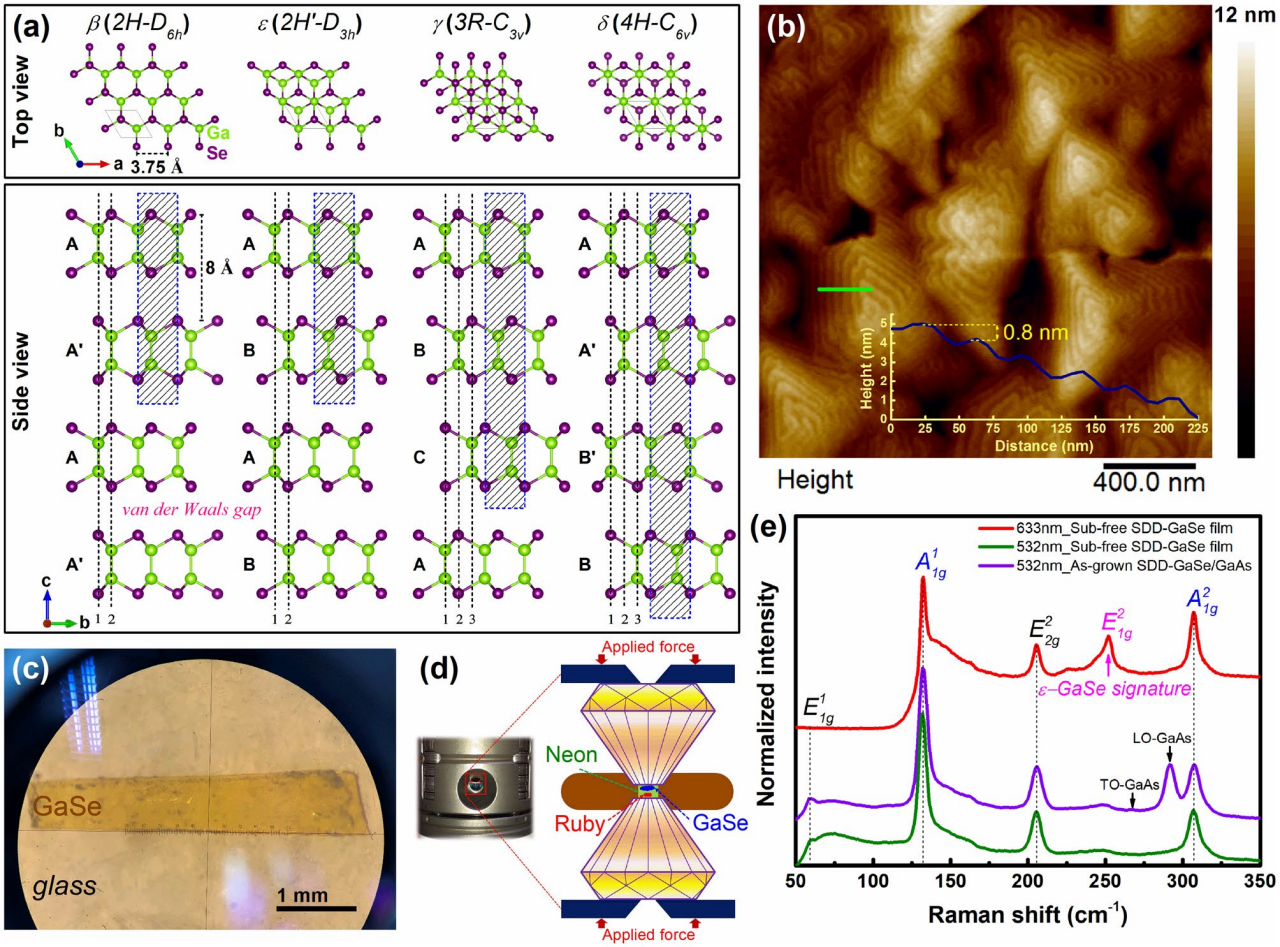
**(a)** Polytypic crystals of 2D GaSe materials visualized by VESTA program^32^. **(b)** AFM image of *ε*-SDD-GaSe grown on GaAs (001) substrate by MBE; inset of **(b)** is step-height profile corresponded to the green line. **(c)** Optical image of a free-standing SDD-GaSe film on glass after substrate removal. **(d)** Illustration of a diamond anvil cell used in the experiment. (**e**) Raman spectra of the SDD-GaSe film before and after substrate removal under 532 nm and 633 nm laser excitation.

The substantial progress of two-dimensional van der Waals (2D-vdW) diatomic-layered GaSe materials in size/thickness scalability, phase/growth mode controllability, and heterostructural/compositional engineering demand more profound investigations^1–3^ to explore a gem of physical properties for interdisciplinary applications from electronics^4,5^, opto-electronics^6–9^, photonics^10–12^, and spintronics^13–15^, to gas sensors^16^, photovoltaic^17^, and water splitting^18–20^. In general, these entire novel behaviors are originated naturally from the diverse electron correlations in weak-bonding vdW interlayers, leading to the complex electronic band structure of the materials. Different from the monotypic graphene, the natural structure of 2D-layered GaSe consists of covalent-bonded Se-Ga-Ga-Se intralayers linked to each other by weak-vdW bonding. The difference in stacking sequences of the intralayers along out-of-plane direction enables thus its phase diversity (including *β*-, *ε*-, *γ*-, and *δ*-phase as illustrated in Fig. 1). This scenario is much more complicated to be understandable and controllable as layer confinement, strain engineering, and structural phase transition are taken into consideration; however, this also opens lots of opportunities to explore physically emergent properties for applications. Indeed, the physical properties of 2D-GaSe material can be experimentally tuned via several approaches, including growth mode^21,22^, substrate alternation^23^, intercalation^24^, layer confinement^25^, strain engineering^26^, oxygen functionalization^27^, and pressurization^28,29^. Among these, employing the high-pressure technique offers a wider degree of freedom to modify the lattice dynamic and thus the interlayer interactions that allow driving the quantum-phase behaviors in 2D materials^30^. In particular, a semiconducting-to-metallic (or even superconducting at low temperature) transition which directly relating to the structural phase transition in *ε*-GaSe bulk was observed at pressure over 25 GPa^28,29,31^. Interestingly, *ε*-GaSe bulk has been also predicted to have a phase crossover from semiconducting to topological insulating at an appropriate biaxial tensile strain of 8% or an equivalently applied pressure of 4 GPa^14,15^. Recently, we have first time reported on the screw-dislocation-driven (SDD) growth mode of 2D-layered GaSe using molecular beam epitaxy (MBE)^22^. In this growth mode, although the pure *ε*-GaSe layered structure was confirmed, its band gap exhibited a strong redshift as compared to that of the bulk counterpart (~ 0.3 eV), which was mostly attributed to the lattice misalignment-induced strain near the screw-dislocation-core (SDC) regions and the substrate–layer interface-correlation^22,23^. Thus, these raise an important question on how the structural and optical properties of a free-standing SDD-GaSe layer grown by MBE, i.e., only the effect of SDCs takes into account, respond to the gradual pressurization. The answer to this would not only introduce a new candidate, SDD-GaSe, for developing future pressure-manipulated electronics or optoelectronics but also be extendible to other 2D materials.

In this study, hydrostatically pressure-driven structural phase transition in a 200 nm substrate-free (sub-free)/free-standing SDD-*ε*-GaSe thin film and GaSe bulk have been characterized comprehensively by in-situ angle-dispersion synchrotron X-ray diffraction (ADXRD) and Raman spectroscopy. Compared with bulk GaSe, an earlier onset point of transition was observed in the SDD-GaSe film at ~ 20 GPa. Remarkably, while the out-of-plane incompressibility of SDD-GaSe film was approximately four times higher than that of the bulk form, a reversible pressure-induced structural transition was evident from the SDD-GaSe film but not from the GaSe bulk. As a result, the impact of SDCs in these phenomena is noticeable and discussed.

## Results and discussion

2D-GaSe has a honeycomb-like structure from the top-view, while a monolayer GaSe is composed of sandwiched links between two Ga atoms and two Se atoms along the side-view (Fig. 1a). For bulk GaSe, there are four different polytypes including *β*-(2H/D_6h_), *ε*-(2H’/D_3h_), *γ*-(3R/C_3v_), and *δ*-(4H/C_6v_), identified by AA’…, AB…, ABC…, and AA’B’B… stacking sequence of consecutive monolayers, respectively^33^. Experimentally, both *ε*-GaSe and *γ*-GaSe are generally co-existent in most of the epitaxial films and their bulk counterpart. In this work, high purity of 200 nm-thick single-crystalline 2D *ε*-GaSe thin films have been epitaxial deposited on GaAs (001) substrate by MBE for the high-pressure experiments. The growth conditions can be referred to our previous work^22^. As shown in Figs. 1b and S1a, the surface morphology of the epitaxial GaSe film grown under SDD growth mode typically exhibited in spiral-like structure. The samples were then mechanically polished to remove the GaAs substrate (Fig. 1c) and carved up to the appropriate sizes for loading into the DAC (Fig. 1d). Raman spectra of the GaSe film before and after substrate removal as shown in Fig. 1e confirm the success of the polishing process based on the elimination of the longitudinal-optical (LO) phonon at 291 cm^−1^ and transverse-optical (TO) phonon signal at 269 cm^−1^ of GaAs substrate. To confirm the dominant phase existing in the SDD film, Raman spectrum of the sub-free GaSe film were also carried out under 633 nm laser excitation. As can be seen in Fig. 1e, the emergence of the ${E}_{1g}^{2}$ Raman resonant mode (~ 251 cm^−1^) under 633 nm laser excitation that was unobservable under 532 nm excitation identifies the dominance of single *ε*-phase in the SDD-GaSe film^22^. Besides, no Raman peak shifting between the samples with and without substrate was observed, meaning that the localized strain potential generated at the film/substrate interface is negligible. High uniformity of the film quality was verified on a sizable area sub-free 2D *ε*-GaSe film (up to ~ 4 mm^2^) as revealed in Fig. S1b. This is also a valuable achievement of our work, offering flexible transferring to other substrates and scalable fabricating opto-electronic devices based on 2D-GaSe materials.

### Anomalous out-of-plane incompressibility and structural transition in SDD-GaSe film

**Figure 2:**
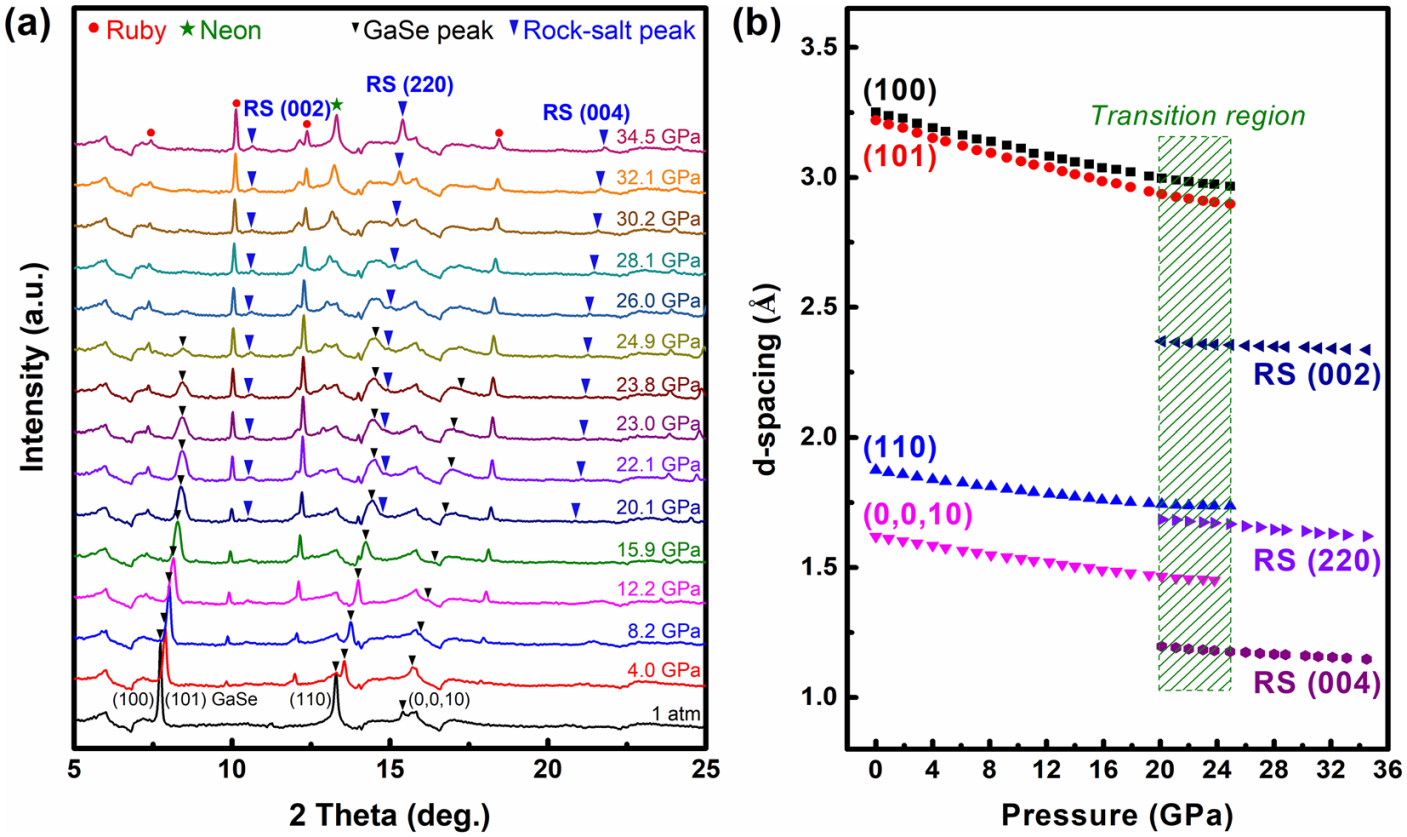
**(a)** Selected ADXRD spectra of the sub-free SDD-GaSe film during pressurization. **(b)** Refined d-spacing of the sub-free SDD-GaSe film as a function of applied pressure.

In order to verify the potential structural phase transition of the materials at high pressure, two parallel in-situ synchrotron ADXRD experiments were carried out on the 200 nm-thick free-standing *ε*-GaSe film and the GaSe bulk under hydrostatic pressurization at room temperature. The compressive pressure ranges for both samples were extended up to 35–40 GPa with fine increments of 0.5 GPa. Selected ADRXD patterns during compression are extracted from 2D-plate images (see Fig. 2a), where diffraction peaks of the SDD-*ε*-GaSe film are displayed (black arrows). As increasing the compression, all GaSe XRD peaks progressively shifted toward higher 2θ-angles along with broadening and diminishing in their linewidth and intensity, respectively. This is attributed to a gradual shrinkage of the crystal lattice with pressure. Indeed, refined d-spacing of the assigned diffraction planes of the GaSe film with pressure exhibits a compression as illustrated in Fig. 2b. As increasing the external pressure beyond ~ 20 GPa, foreign features located at 2θ angles of ~ 10.4°, ~ 14.8°, and ~ 20.9° started to be visible, while all hex-GaSe lines were continuously being suppressed and substantially disappeared at ~ 25 GPa. In comparison, the foreign features in the bulk appeared later than that in the GaSe film where its onset pressure is ~ 22 GPa (Fig. S2). The rise of alien diffractions is a signature of the structural phase transition from the hexagonal-to-rock salt (hex-to-RS) phase as reported previously^29,34^, where these XRD film peaks (blue arrows) can be assigned as (002), (220), and (004) diffraction lines of the GaSe rock-salt phase.

To give a better understanding of the structural transition on the epitaxial film and bulk sample, we have analyzed the unit-cell volume (*V*) and the normalized cell parameters *a(P)/a*_*0*_, *c(P)/c*_*0*_, *c(P)/a(P)*, and *V(P)/V*_*0*_ as a function of pressure, where *a*_*0*_, *c*_*0*_, and *V*_*0*_ are in-plane, out-of-plane lattice constant, and unit-cell volume at ambient conditions, respectively. Then, the individual lattice compressibility along *a*-axis and *c*-axis as well as the bulk modulus of the samples were quantitated using inverted Birch-Murnaghan Equation of State (BM-EoS) fitting as described in Eqs. (1–3)^35,36^:

1 $$\frac{a(P)}{{a}_{0}}={\left[1+\frac{{K}_{0a}^{^{\prime}}}{{K}_{0a}}P\right]}^{-\frac{1}{{K}_{0a}^{^{\prime}}}}$$

2 $$\frac{c(P)}{{c}_{0}}={\left[1+\frac{{K}_{0c}^{^{\prime}}}{{K}_{0c}}P\right]}^{-\frac{1}{{K}_{0c}^{^{\prime}}}}$$

and

3 $$\frac{V(P)}{{V}_{0}}={\left[1+\frac{{B}_{0}^{^{\prime}}}{{B}_{0}}P\right]}^{-\frac{1}{{B}_{0}^{^{\prime}}}}$$

where *K*_*0a*_ (*K*^*’*^_*0a*_) and *K*_*0c*_ (*K*^*’*^_*0c*_) are in turn in-plane (IP) and out-of-plane (OOP) inverse linear compressibility (its pressure derivative). Hereinafter *K*_*0a*_ and *K*_*0c*_ are called as IP and OOP incompressibility, respectively. *B*_*0*_ (*B*^*’*^_*0*_) is the bulk modulus (its pressure derivative) of the materials.

**Figure 3:**
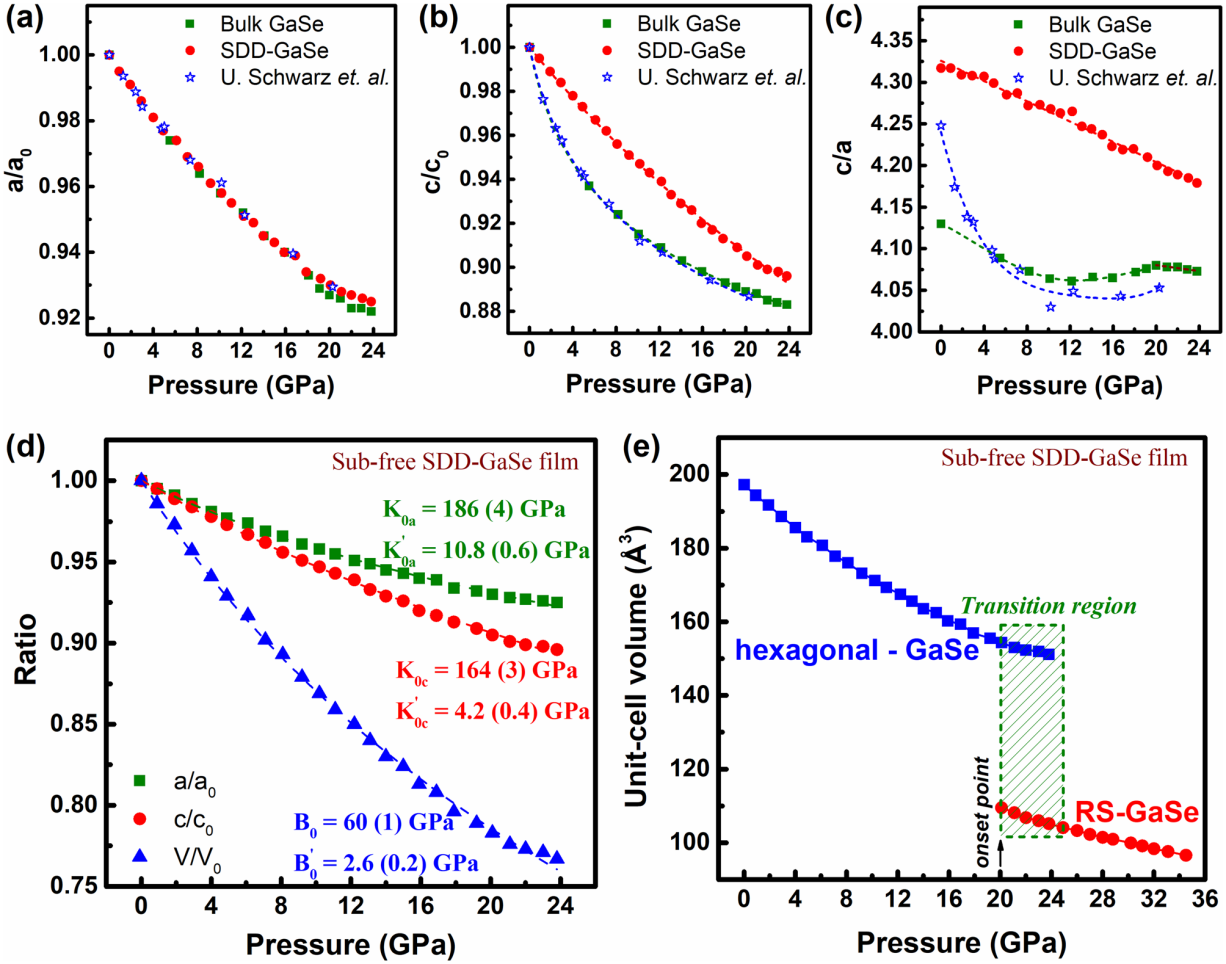
**(a–c)** Pressure dependence of normalized lattice parameters of the sub-free SDD-GaSe film and GaSe bulk, in comparison to the reproduced data with permission from Ref.^29^. **(d)** Inverted Birch-Murnaghan EoS fitting of the sub-free SDD-GaSe film. **(e)** Unit-cell volume of the sub-free SDD-GaSe film as a function of pressure revealing a transition region (20–25 GPa) from hexagonal to high-pressure rock-salt phase.

The results are addressed in Fig. 3 and Table 1, including reproduced experimental data on GaSe bulk from U. Schwarz’s group for reference^29^. Figure 3a displays an excellent agreement in the pressure-dependent in-plane lattice constant (*a*) between our bulk sample and the referred study. Interestingly, even though the sub-free-SDD-GaSe film possesses a distinct layer-stacking configuration, i.e., spiral stacking, as compared to the layer-by-layer (LBL) stacking in bulk samples^22^, the pressure-dependent in-plane parameters observed in the GaSe film and the bulk samples are no visual difference. Indeed, the *IP* incompressibility values of these samples are comparable, which are ~ 186 ± 7 GPa (for sub-free SDD-GaSe film), 188 ± 7 GPa (GaSe bulk), and 198 ± 10 GPa (referred data). In other words, regardless of different layer stacking configurations, the in-plane compressibility in 2D-GaSe materials is identical. In contrast, the pressure-dependent out-of-plane lattice parameter of the SDD-GaSe film reveals an unusual phenomenon as shown in Fig. 3b,c.

**Table 1:** Incompressibility parameter and bulk modulus of the SDD-GaSe film and GaSe bulk extracted from the inverted Birch-Murnaghan EoS.

Sample	In-plane incompressibility (GPa)		Out-of-plane incompressibility (GPa)		Bulk modulus (GPa)		Investigated pressure range
	*K*_*0a*_	*K*^*’*^_*0a*_	*K*_*0c*_	*K*^*’*^_*0c*_	*B*_*0*_	*B*^*’*^_*0*_	
GaSe film	186 ± 4	10.8 ± 0.6	164 ± 3	4.2 ± 0.4	60 ± 1	2.6 ± 0.2	0–35 GPa
GaSe bulk	188 ± 6	9.3 ± 1	40.5 ± 0.7	20.6 ± 0.2	35.5 ± 1	5.1 ± 0.2	0–40 GPa
Ref.^29^	198 ± 10	9 ± 2	44 ± 2	18.7 ± 7	34 ± 2	6.4 ± 5	0–39 GPa

Its compression rate was nearly linear and much lower than that of the bulk samples, especially at the low-pressure range of < 5.0 GPa. In fact, from *c(P)/c*_*0*_ inverted BM-EoS fitting, while the *OOP* incompressibility of our GaSe bulk is in good agreement to the data of Schwarz et al.^29^, the *K*_*0c*_ of SDD-GaSe film unveils a notable value of 164 ± 5 GPa, i.e., ~ 4 times higher in magnitude than those of the bulk samples. We claim that the classically spiral structure in SDD-GaSe film is much more incompressible and strongly close-packed along *c*-axis, which may be due to building up in coupling interaction between adjacent GaSe intra-layers, especially near SDCs. This behavior thus directly results in a ~ 1.7 times higher bulk modulus of the SDD-GaSe film (*B*_*0f.*_ ~ 60 ± 4 GPa) as compared to that of the bulk sample (*B*_*0*_^*b*^ ~ 35.5 ± 3 GPa) as noted in Figs. 3d and S3a. Furthermore, abrupt shrinkages in the unit-cell volume (*V*) that were observed at specific pressures, i.e., ~ 20 GPa for the SDD-GaSe film (Fig. 3e) and ~ 22 GPa for the GaSe bulk (Fig. S3b), are identified as the onset point of high-pressure GaSe RS phase^29,34^. It is noticed that the earlier RS onset point in the SDD-GaSe film could be subject to owning a large amount of SDCs in its unique crystalline structure and will be discussed later. Despite that, either the SDD-GaSe film or the GaSe bulk exhibits an equivalent end-point of the hex-to-RS structural phase transition (indicated by the completely quenching of GaSe XRD peaks) at ~ 25 GPa, which may be concurrent with the semiconducting-to-metallic electronic phase transition of the material^28^.

### Optical phonon shifting under pressure in SDD-GaSe film

**Figure 4:**
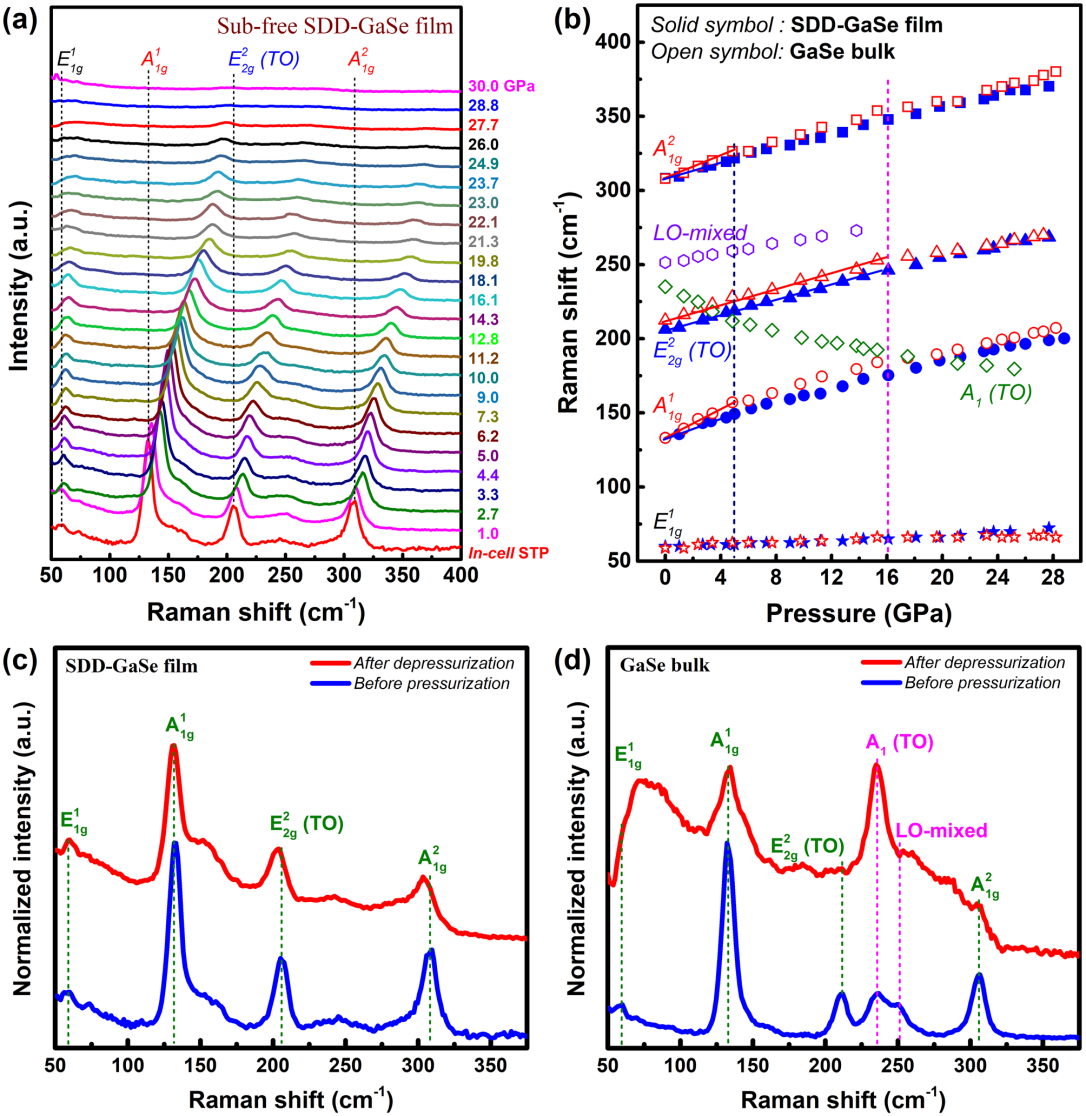
**(a)** Selected Raman spectra under 532 nm laser excitation of the sub-free SDD-GaSe film during pressurization. **(b)** Pressure dependence of phonon vibration modes observed from the sub-free SDD-GaSe film and the GaSe bulk. STP: standard temperature pressure at ambient. 532 nm excited Raman spectra of **(c)** the sub-free SDD-GaSe film and **(d)** the GaSe bulk before pressurization and after depressurization.

In-situ high-pressure Raman scattering measurement is also a powerful approach for gaining insight of the structural phase transition of the material via determining its phonon vibrational variation during compression^31,37^. Thus, this characterization was employed in this study on both samples. Due to the major *ε*-phase in the free-standing SDD-GaSe epitaxial film^22^, its crystal structure belongs to the non-centrosymmetric ${D}_{3h}^{1}$ group. Theoretically, it has 24 normal modes at the *Γ* point of the Brillouin zone, which is consisted of 11 non-degenerate Raman active modes, 6 non-degenerate IR active modes, and 2 acoustic vibration modes^38,39^. Figure 4a shows selected Raman spectra of the SDD-GaSe film during the experimental pressurization, where four typical phonon vibration modes of *ε*-GaSe, located at ~ 59.9 cm^−1^ (assigned to ${E}_{1g}^{1}$); ~ 133.2 cm^−1^ (${A}_{1g}^{1}$); ~ 206.4 cm^−1^ (${E}_{2g}^{2}$), and ~ 308.3 cm^−1^ (${A}_{1g}^{2}$) have been observed at in-cell ambient pressure.

As increasing the applied pressure, all of these modes (either IP or OOP vibration modes) gradually shifted towards the higher frequencies accompanied with their broadening in linewidth and deteriorating in intensity; and finally, were almost undetectable at ~ 29 GPa. It means that the layered structure of SDD-GaSe film was undergoing a great shortening not only in the vdW-interlayer gaps but also in the atomic distances in the intralayers, resulting in a visible lattice compression. Qualitatively, this behavior is well-consistent with what was observed from pressure-dependent ADXRD data presented above. To have a deeper insight, we plot the evolution of phonon vibration modes in the SDD-GaSe film and the bulk versus pressure as shown in Fig. 4b. The extracted pressure coefficient (defined by *δ* = *dω/dP*) and Grüineisen parameter of each Raman modes are also tabulated in Table 2.

**Table 2:** Experimental ambient-pressure frequency (*ω*_*0*_), pressure coefficient (*δ*), and Grüineisen parameter ($\overline{\gamma }$) of the Raman active modes observed from the SDD-GaSe film and the GaSe bulk.

Mode parameter	${\omega }_{0}$ (cm^−1^)		$\delta = \frac{d\omega }{dP}$ (cm^−1^/GPa)		$\overline{\gamma }= \frac{1}{{\omega }_{0}}\frac{d\omega }{dP}$ (× 10^–3^ GPa^−1^)		Pressure range
	Film	Bulk	Film	Bulk	Film	Bulk	
${E}_{1g}^{1}$	59.9	58.8	0.41 ± 0.02	0.26 ± 0.02	6.8 ± 0.3	4.4 ± 0.4	0–28 GPa
${E}_{2g}^{2} (TO)$	206.4	212.4	2.51 ± 0.04	2.35 ± 0.07	12.2 ± 0.2	11.1 ± 0.3	0–16 GPa
			2.31 ± 0.03	2.04 ± 0.06	11.2 ± 0.2	9.6 ± 0.3	0–28 GPa
${E}_{1g}^{2}/{A}_{1} (LO)$		251.4		1.58 ± 0.03		6.3 ± 0.1	0–16 GPa
${A}_{1g}^{1}$	133.2	133.1	**3.26** ± 0.18	**4.96** ± 0.18	24.5 ± 1.4	37.3 ± 1.4	0–5 GPa
			2.33 ± 0.04	2.41 ± 0.09	17.5 ± 0.3	18.1 ± 0.7	0–28 GPa
${A}_{1g}^{2}$	308.3	308.1	**2.78** ± 0.16	**3.85** ± 0.17	9.0 ± 0.5	12.5 ± 0.5	0–5 GPa
			2.29 ± 0.03	2.42 ± 0.06	7.4 ± 0.1	7.9 ± 0.2	0–28 GPa
${A}_{1} (TO)$		235.1		**− 4.84** ± 0.19		− 20.6 ± 0.8	0–5 GPa
				− 2.09 ± 0.14		− 8.9 ± 0.6	0–28 GPa

The bold values indicate the highlighted pressure coefficients of out-of-plane Raman active modes in the SDDGaSe film and bulk at a low-pressure range (< 5 GPa).

There are two noteworthy points from our analyses on SDD-GaSe film. Firstly, the ${E}_{2g}^{2}$ mode in the sub-free SDD-GaSe film at ambient pressure exposed an obvious redshift of 6.0 cm^−1^ as compared to that in the GaSe bulk. This corresponds to the unintentional appearance of both in-plane and out-of-plane strains induced by SDD-growth mode^22,23^. Even that, similar to the GaSe bulk, this vibration mode revealed a unity in pressure coefficient (*δ* ~ 2.4 cm^−1^/GPa) as the pressure increased up to ~ 16 GPa. This result is well-matched to the equivalency in IP incompressibility between SDD-GaSe film and GaSe bulk as extracted from the ADXRD data. Interestingly, above 16 GPa, the ${E}_{2g}^{2}$ frequency of SDD-GaSe film was getting close to that of GaSe bulk, and then those almost coincided with each other at pressures above 20 GPa. Thus, this tends to indicate a similar degree of compression in both SDD-GaSe film and GaSe bulk. Secondly, as observing the out-of-plane vibration modes of the SDD-GaSe film, i.e., ${A}_{2g}^{1}$ and ${A}_{2g}^{2}$ modes, their pressure-dependent behaviors could be noticed in two regions (Fig. 4b). In the first region (low-pressure range $\le$ 5 GPa), both of these modes showed a lower blue-shifted rate than in the bulk’s one, corresponding to a smaller SDD-GaSe film’s *δ* value (Table 1). In other words, the lattice compression along *c*-axis at the low-pressure range is less pronounced in the SDD-GaSe film than in the bulk. Again, this statement is correlated to the high OOP incompressibility of the GaSe film (Fig. 3d). In the later region (> 5 GPa), the pressure coefficients of these modes in both GaSe film and bulk were approaching together since the lattice is less compressible when it is more and more close-packed.

It is necessary to take into account the simultaneous existence of *ε*- and *γ*-phase in the GaSe bulk grown by the Bridgman method^40^, as manifested by the appearance of extra broadband with centers located at ~ 235.1 cm^−1^ [${A}_{1}(TO)$] and ~ 251.4 cm^−1^ [could be a LO-mixed mode of ${A}_{1}(LO)$ at ~ 247 cm^−1^ and ${E}_{1g}^{2}(LO)$ at ~ 253 cm^−1^] in Raman spectrum of the GaSe bulk at ambient pressure (Fig. S4)^39^. As increasing the applied pressure, the LO-mixed mode monotonously shifted to the higher frequencies with a smaller pressure coefficient *δ* as compared to that of ${E}_{2g}^{2}(TO)$, then is almost undetectable at ~ 16 GPa. On the other hand, the ${A}_{1}(TO)$ peak, which is considered as a signature of *γ*-GaSe^39,41^, exhibited an abnormal trend upon compression by nonlinearly shifting to the lower frequencies in a wide range of pressure up to ~ 25 GPa, and then disappeared at the end-point of the hex-to-RS phase transition. The observed negative pressure coefficient of the $\gamma -{A}_{1}(TO)$ mode could be explained well when the competition between interlayer interaction and Ga-Se chemical bond-tilting effect along with the atomic vibration direction of three observed OOP modes (Fig. S4b) are taken into consideration. For the ${A}_{1g}^{1}$ mode, two Se atoms in an intralayer vibrate out-of-phase to each other, whereas Ga and Se atom vibrations are in-phase. Thus, this mode is only governed by the strong interlayer interaction since the bond-tilting effect is negligible, showing a high positive *δ* (see bold values in Table 2). Different from ${A}_{1g}^{1}$ mode, these effects are visible (out-of-phase vibration presented in either Se/Se or Ga/Se pair) and compensate each other in the ${A}_{1g}^{2}$ mode, leading to a smaller positive *δ*. On the contrary, the interlayer interaction could be ignored in the $\gamma -{A}_{1}(TO)$ mode due to the in-phase vibration between Se atoms in each intralayer as well as interlayer, while the Ga/Se out-of-phase vibration could strongly promote the bond-tilting effect, resulting in the negative *δ* of this mode. Moreover, the pressure-induced redshift phonon frequency could be a characteristic of the TO-LO splitting due to the long-range Coulomb interaction. This behavior was also observed in some Se-based 3D-conventional semiconductors such as ZnSe, ZnFeSe, and ZnMnSe^42,43^.

### Unique structural phase reversibility in SDD-GaSe film

After compressing the material up to ~ 35 to 40 GPa, the samples were gradually decompressed back to ambient pressure. The down-stroke Raman spectra of both SDD-GaSe and the bulk samples are shown in Fig. S5. In the case of SDD-GaSe film, it seems likely that the non-polar out-of-plane vibration mode-${A}_{1g}^{1}$ was revived at the very early stage of the depressurization, then gradually redshifts, and being pronounced at ~ 4.8 GPa along with the appearance of the signal from in-plane vibration mode (${E}_{2g}^{2}$). On the other hand, the down-stroke spectra starting from 30 GPa of the bulk sample revealed the gentle revitalization of a broadband relating to ${A}_{1g}^{1}$ and ${A}_{1}(TO)$ modes. Interestingly, after completely releasing external pressure, the Raman spectrum of SDD-GaSe film exhibited a reversible property, where all SDD-GaSe Raman active modes were well-recovered (Fig. 4c). The enhancement of a broad-peak located at ~ 155 cm^−1^ may be due to the laser-induced oxidation after the long time-experiment. Besides, the slight redshifts of all Raman features are attributed to the distorted lattice structure after compression. On the contrary, the decompressed Raman spectrum of bulk-GaSe is irreversible with the dominance of *γ*-phase signal at the end (Fig. 4d). These results might be due to the differences in the high-pressure structural phase transition mechanism between the Bridgman-grown GaSe bulk and the MBE-grown SDD-GaSe film. We claim that the bulk sample may undergo the layer-sliding process upon compression, while the SDCs play a major factor in promoting the phase transition in SDD-GaSe under high pressure.

### Role of screw dislocation cores

**Figure 5:**
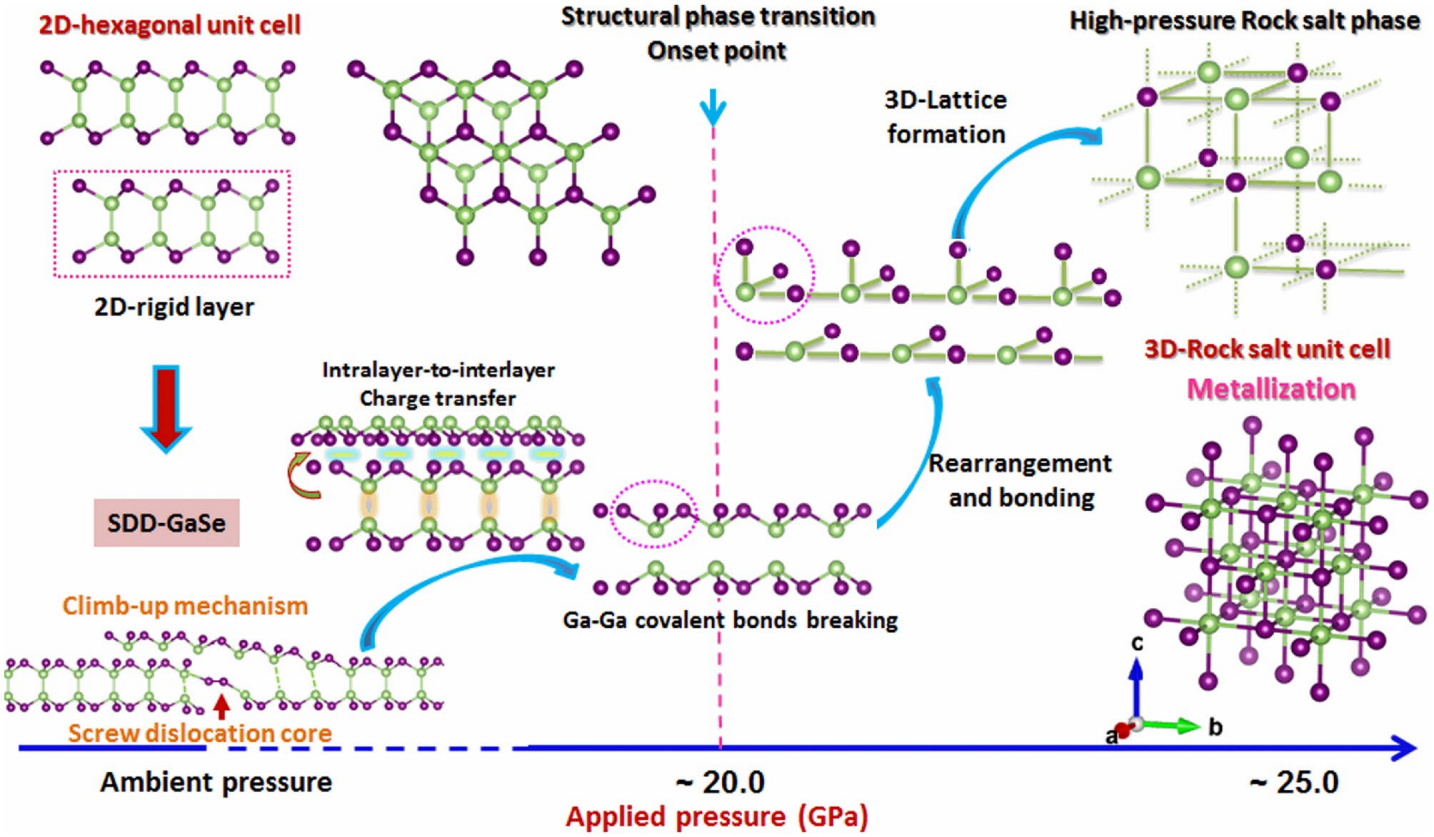
A summarized evolution of the structural phase transition occurring in a free-standing SDD-GaSe film under external pressure.

Previous researches have shown that because of the typical LBL-vdW structure of most 2D materials, they have possibly experienced the layer sliding under high pressure^44,45^. In agreement with our experimental findings as shown in the ADXRD data, the slope changing (from negative to positive) in the GaSe bulk *c/a* profile (Fig. 3c) at ~ 16 GPa is said to be evidence of pressure-induced layer sliding, leading to the alteration in the layer-stacking configuration. As looking back on Fig. 1a, the difference in stacking configuration between *γ* and *ε*-GaSe phase is only the horizontal translation of the third rigid-layer (noted by layer C). Thus, it is a possible occurrence of *ε*-to-*γ* phase transition due to the layer-sliding effect during pressurization. Indeed, in the LBL-GaSe bulk sample, the LO-mixed mode almost disappeared at ~ 16 GPa, whereas the ${A}_{1}(TO)$ mode that belonged to *γ*-phase could survive up to ~ 25 GPa (Figs. 4b and S4a). This means that the high-pressure (HP) phase transition in the case of LBL-GaSe bulk could include a *ε*-to-*γ* phase transition, followed by the hex-to-RS structural transition. In light of these observations, we suppose that the pressure-induced *ε*-to-*γ* phase transition via the layer-sliding phenomenon in the LBL-GaSe bulk required a large amount of activation energy, which could be the main reason for the phase irreversibility of the LBL-GaSe bulk after decompression as mentioned above. On the other hand, the MBE-grown SDD-GaSe film is supposed to undergo a distinctive pressure-induced structural phase transition mechanism, where the SDCs play a major driving force in the hex-to-RS transformation and vice versa. To describe visibly the scenario, we make a high-pressure structural phase transition diagram of SDD-GaSe as shown in Fig. 5. Firstly, our previous study has demonstrated that the growth of SDD-GaSe is hypothesized to experience an edge climb-up process at SDCs, where the Se-Se inter-edge dimerization was taking place (Fig. S6), resulting in the discontinuity in the atomic chain-layer, and hence, enhancing the interlayer interaction^46^. As a result, the SDD-GaSe film is more incompressible along the *c*-axis than that of the LBL-GaSe bulk presented by the four-times larger in the *OOP* incompressibility and lower pressure coefficient *δ* of SDD-GaSe. Furthermore, according to the climb-up mechanism in the SDD-GaSe film, it is acceptable to assume that the intralayer Ga-Ga bonds located near the SDCs are mostly unstable. Secondly, at sufficient applied pressure, the pressure-induced intralayer-to-interlayer charge transfer becomes dominant, leading to further weakening of the Ga-Ga bonding strength^47^. Thus, the SDCs could play as the primers to initiate disruption of the neighbored Ga-Ga covalent-bonding chains, followed by the rearrangement of free half-rigid layers. Finally, the Ga and Se atoms from adjacent half-rigid layers catch each other to form Ga-Se metallic bonds, which is similar to the bonds in the GaSe RS structure. Although the SDD-GaSe film is more incompressible, it seems to be more brittle as compared to the GaSe bulk because of possessing an intensely high-density of SDCs in its lattice structure. The HP phase transition barrier in SDD-GaSe could be smaller than that of the LBL-GaSe structure. In other words, SDCs could assist the hex-to-RS transition to occur earlier, evidenced from the lower RS onset point of SDD-GaSe film compare to that of the bulk. However, it is possible that the SDCs were still being memorized in the RS structure of the SDD-GaSe film because of the strong coupling between adjacent haft-rigid layers near the SDC sites. Therefore, as entirely releasing the sample from the external pressure, these SDCs could now promote the recovery of the original layer-stacking configuration as observed from the well-recuperated *ε*-GaSe Raman modes.

## Conclusions

In summary, pressure-induced structural phase transition of the free-standing SDD-GaSe layer grown by MBE has been comprehensively investigated for the first time by in-situ ADXRD and Raman spectroscopy. According to the results, we found that the physical behaviors under high pressure of SDD-GaSe layer are very distinct from the LBL-GaSe bulk form. The SDD-GaSe film exhibited an earlier onset point of hexagonal-to-rock salt transition, a 4-times higher value in the incompressibility along *c*-axis that leading to a 1.7 times higher bulk modulus in comparison to the LBL-GaSe bulk. Surprisingly, the pristine *ε*-phase structure of the SDD-GaSe film was well-recovered after decompression, whereas the structure of GaSe bulk was irreversible from its high-pressure phase and seems to prefer forming *γ*-phase after depressurization. Thus, we tend toward a statement that these novel phenomena are mainly governed by the large-density SDCs existed in the SDD-GaSe film, which makes it more incompressible along out-of-plane direction but more transformable as well. This work contributes to insight interesting physics of structural phase transition not only in 2D GaSe also other SDD-related 2D materials and pave the way for developing future pressure-manipulated electronics or optoelectronics based on 2D materials.

## Methods

### Materials synthesis and high-pressure preparation

While the GaSe bulk sample was synthesized by the Bridgman method, 200-nm-thick SDD-*ε*-GaSe thin film was grown on semi-insulating GaAs (001) substrate by MBE. The growth conditions of SDD-GaSe film were presented in our previous work^22^. To prepare the free-standing SDD-GaSe films, the sample substrate was carefully removed by mechanical polishing, in which the large lateral size of the sub-free film can be achieved up to ~ 4.0 mm^2^ as shown in Fig. 1b. Then, both GaSe bulk and SDD-GaSe film were carved up into small pieces of ~ 50 × 50 µm^2^ in size and loaded separately into two diamond anvil cells (DACs) with 350 µm culet diameters. For all the experiments, Re gaskets were first indented and then drilled to perform hole-chambers with diameters of 165 µm and thicknesses of 70 µm, while ruby spheres (~ 5 µm in radius) were employed for pressure calibration^48^. The hydrostatic high-pressure measurements were conditioned by using Neon (Ne) gas and a mixture of methanol and ethanol (4:1) as pressure transmitting media for ADXRD and Raman measurements, respectively.

### In-situ ADXRD measurements

In-situ high-pressure ADXRD measurements of the samples were carried out at 13-BMC-GeoCARS beamline of the Advanced Photon Source, Argonne National Laboratory, USA with an incident X-ray beam wavelength of 0.434 Å, where membrane pressure-controlled DAC was employed. An exposure time of 120 s was synchronized for all experiments. The 2D XRD images were collected by a Pilatus1M plate detector and integrated into 1D ASCII files by the Dioptas program^49^.

### In-situ Raman measurements

In-situ high-pressure Raman spectra of the samples were conducted at room temperature by a HORIBA TRIAX 550 spectrometer in the backscattering geometry with an excitation laser wavelength of 532 nm.

## Supplementary Information

Supplementary Figures.
