# Supplementary material for: Pressure induced structural phase crossover of a GaSe epilayer grown under screw dislocation driven mode and its phase recovery
Source: Sci Rep. 2021 Oct 6;11:19887. doi: 10.1038/s41598-021-99419-1 (PMC8494905; doi:10.1038/s41598-021-99419-1)
Supplement: Supplementary file 1 — Supplementary Figures. [file 41598_2021_99419_MOESM1_ESM.docx]

**ELECTRONIC SUPPLEMENTARY INFORMATION for**

**Pressure induced structural phase crossover of a GaSe epilayer grown under screw dislocation driven mode and its phase recovery**

Nhu Quynh Diep^1^, Ssu Kuan Wu^1^, Cheng Wei Liu^1^, Sa Hoang Huynh^1,*^, Wu Ching Chou^1,*^, Chih Ming Lin^2,*^, Dong Zhou Zhang^3^, and Ching Hwa Ho^4^

^1^Department of Electrophysics, College of Sciences, National Yang-Ming Chiao-Tung University, Hsinchu 30010, Taiwan.

^2^Department of Physics, College of Sciences, National Tsing Hua University, Hsinchu 300040, Taiwan.

^3^GeoSoilEnviroCARS, Argonne National Laboratory, Illinois, USA.

^4^Graduate Institute of Applied Science and Technology, National Taiwan University of Science and Technology, Taipei 106, Taiwan.

^*^Corresponding authors: Prof. W. C. Chou ([wcchou957@nycu.edu.tw](mailto:wcchou957@nycu.edu.tw)), Prof. C. M. Lin ([cm_lin@phys.nthu.edu.tw](mailto:cm_lin@phys.nthu.edu.tw)), and Dr. Sa Hoang Huynh ([hoangsa1429@nycu.edu.tw](mailto:hoangsa1429@nycu.edu.tw))


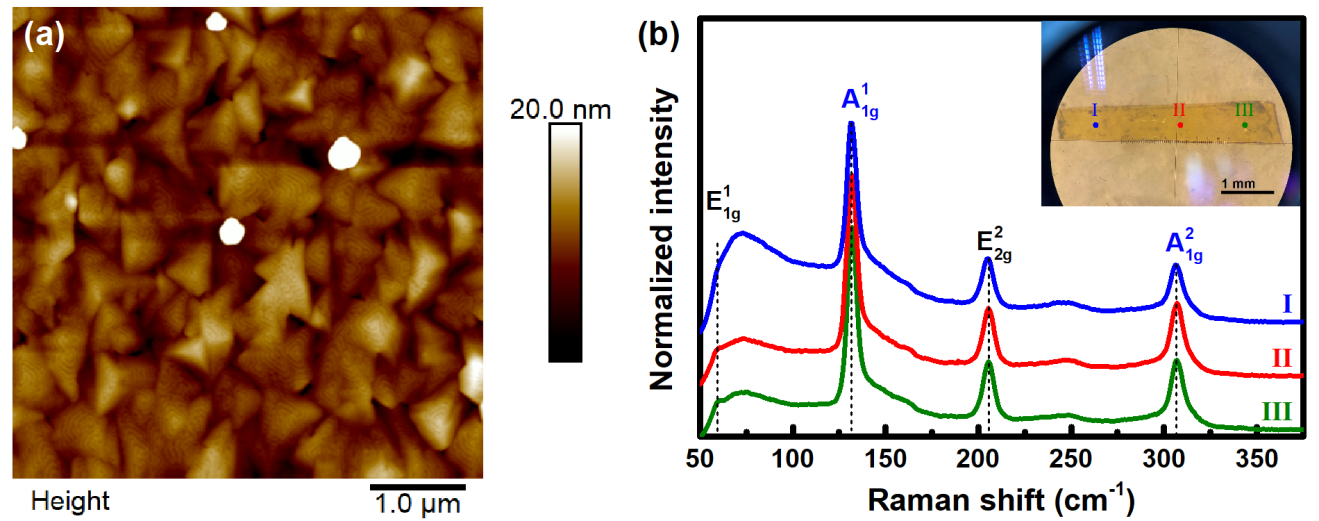


**Figure S1.** (a) Large-scale AFM image of the SDD-GaSe. (b) Raman spectra of sub-free SDD-GaSe film at different positions under 532-nm laser excitation.


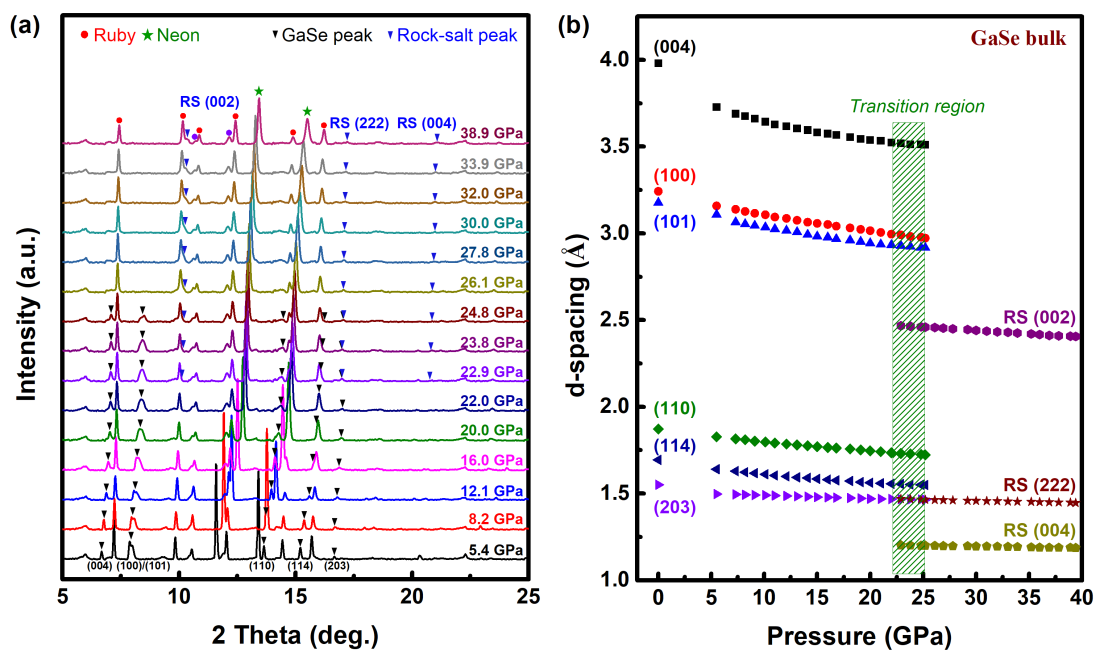


**Figure S2.** (a) Selected ADXRD spectra of the GaSe bulk during pressurization. (b) Refined d-spacing of the GaSe bulk as a function of applied pressure.


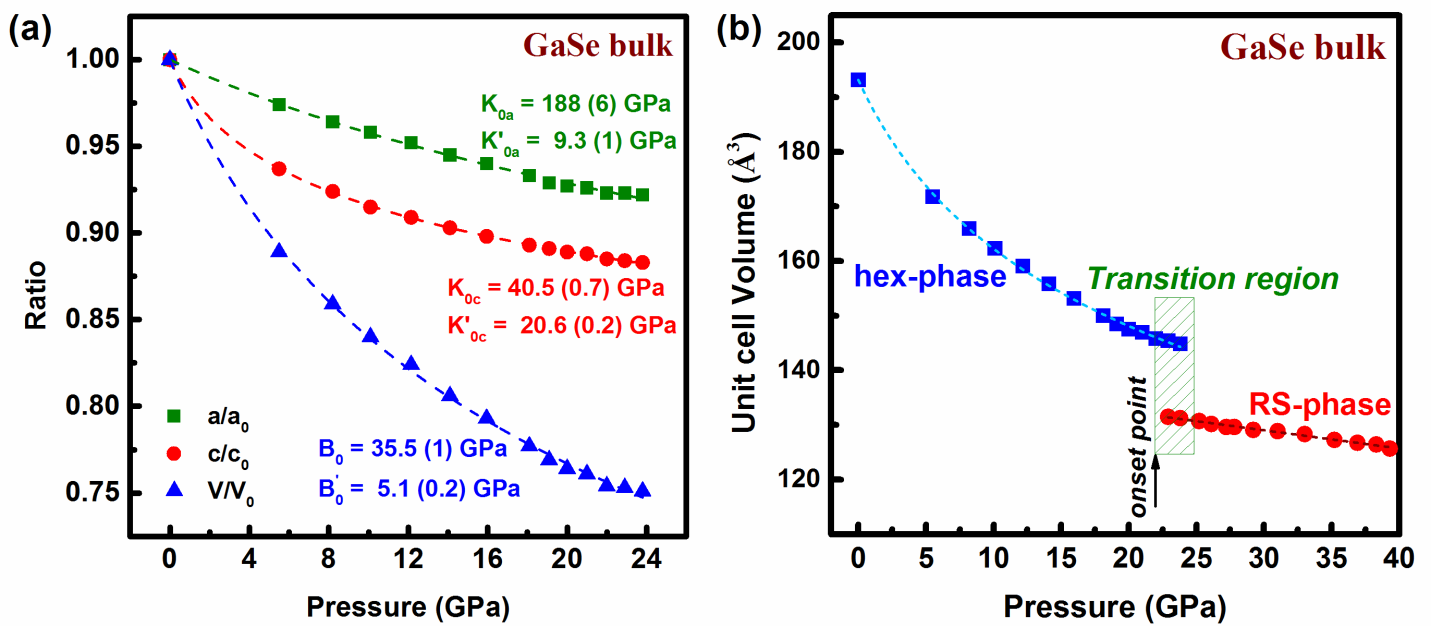


**Figure S3.** (a) Inverted Birch-Murnaghan EoS fitting of the GaSe bulk. (b) Unit-cell volume of the GaSe bulk as a function of pressure revealing a transition region (22 – 25 GPa) from hexagonal to high-pressure rock-salt phase.


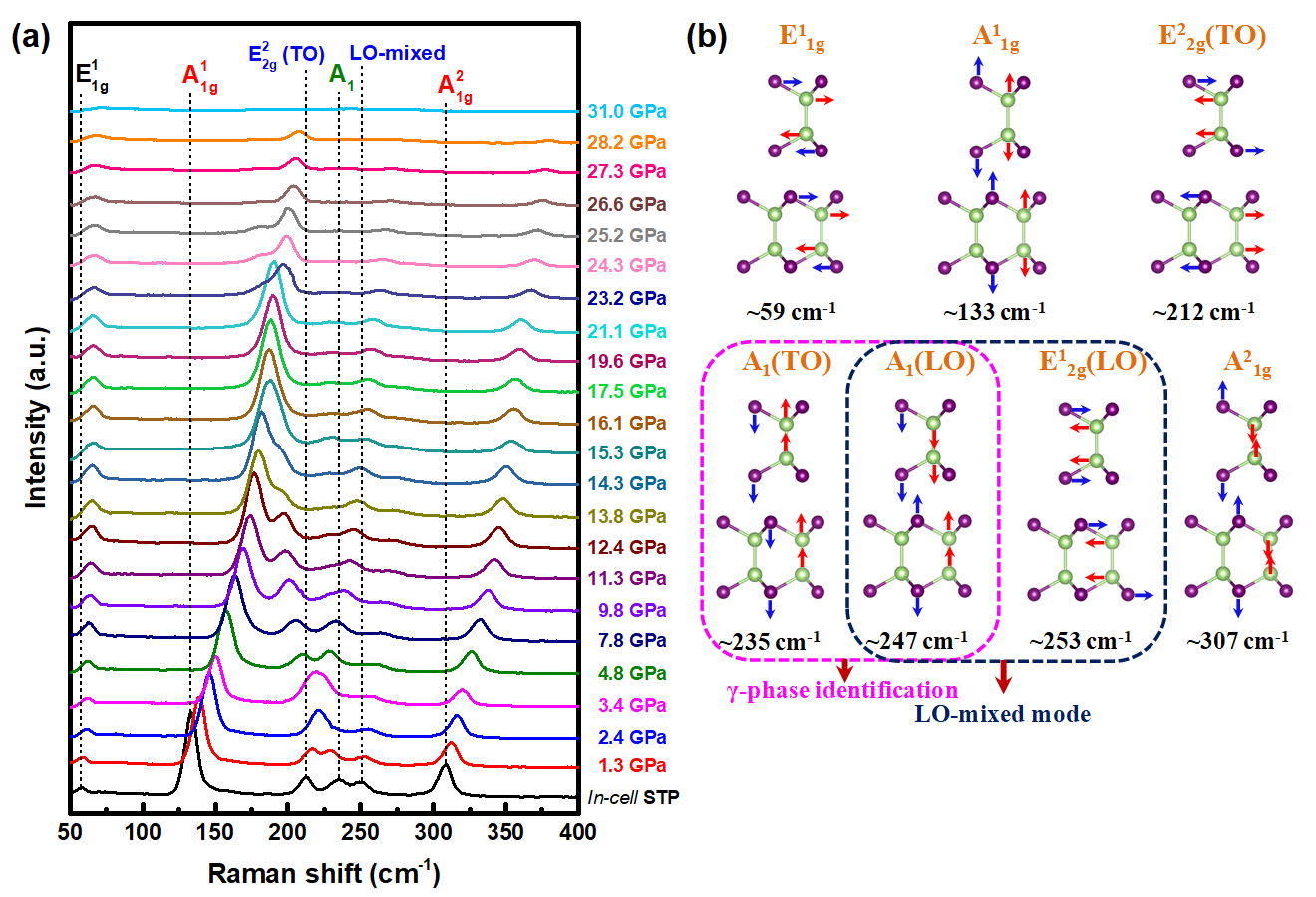


**Figure S4.** (a) Selected Raman spectra under 532 nm excitation of the GaSe bulk during pressurization. (b) Visualized configuration of phonon vibration modes observed from the GaSe bulk.^39^


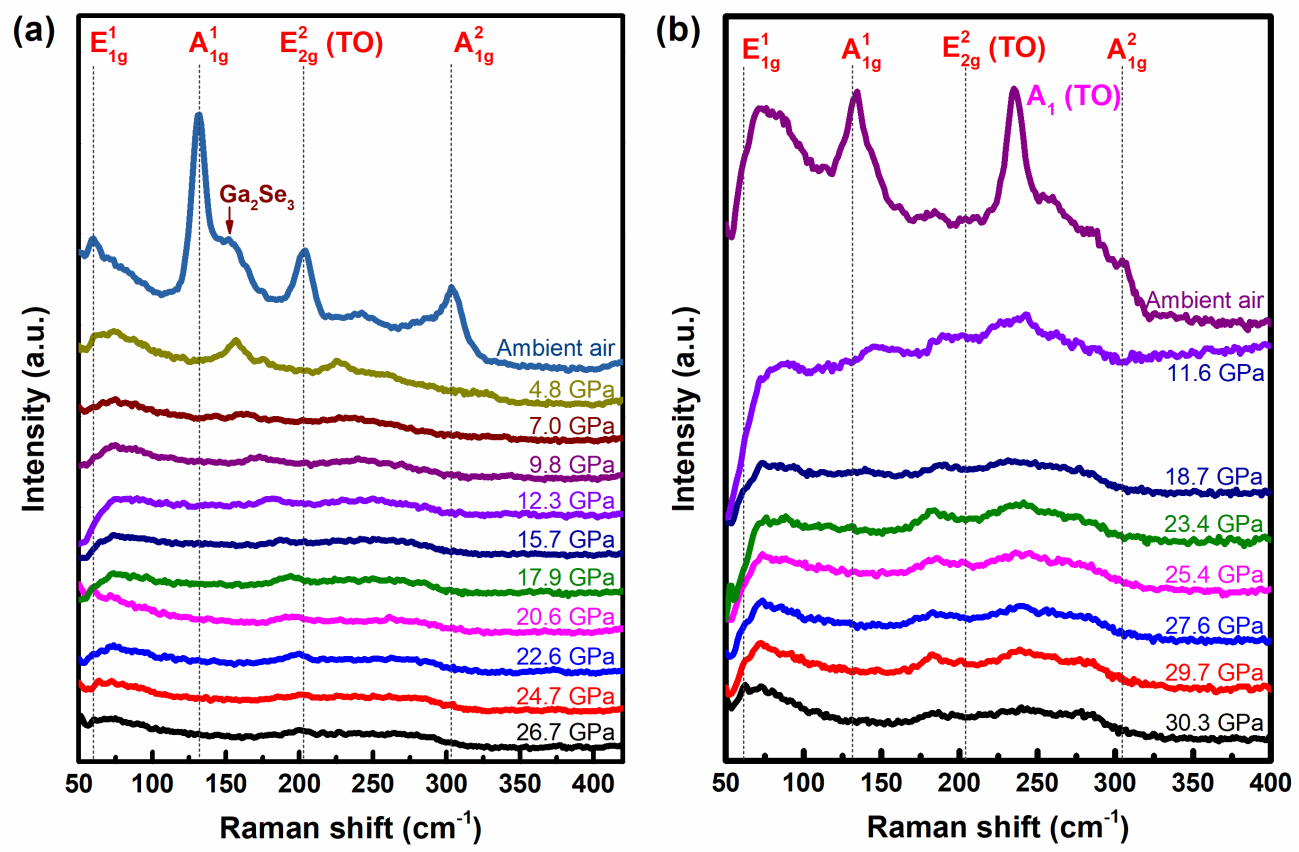


**Figure S5.** Selected Raman spectra under 532 nm laser excitation of (a) the sub-free SDD-GaSe film and (b) the GaSe bulk during depressurization.


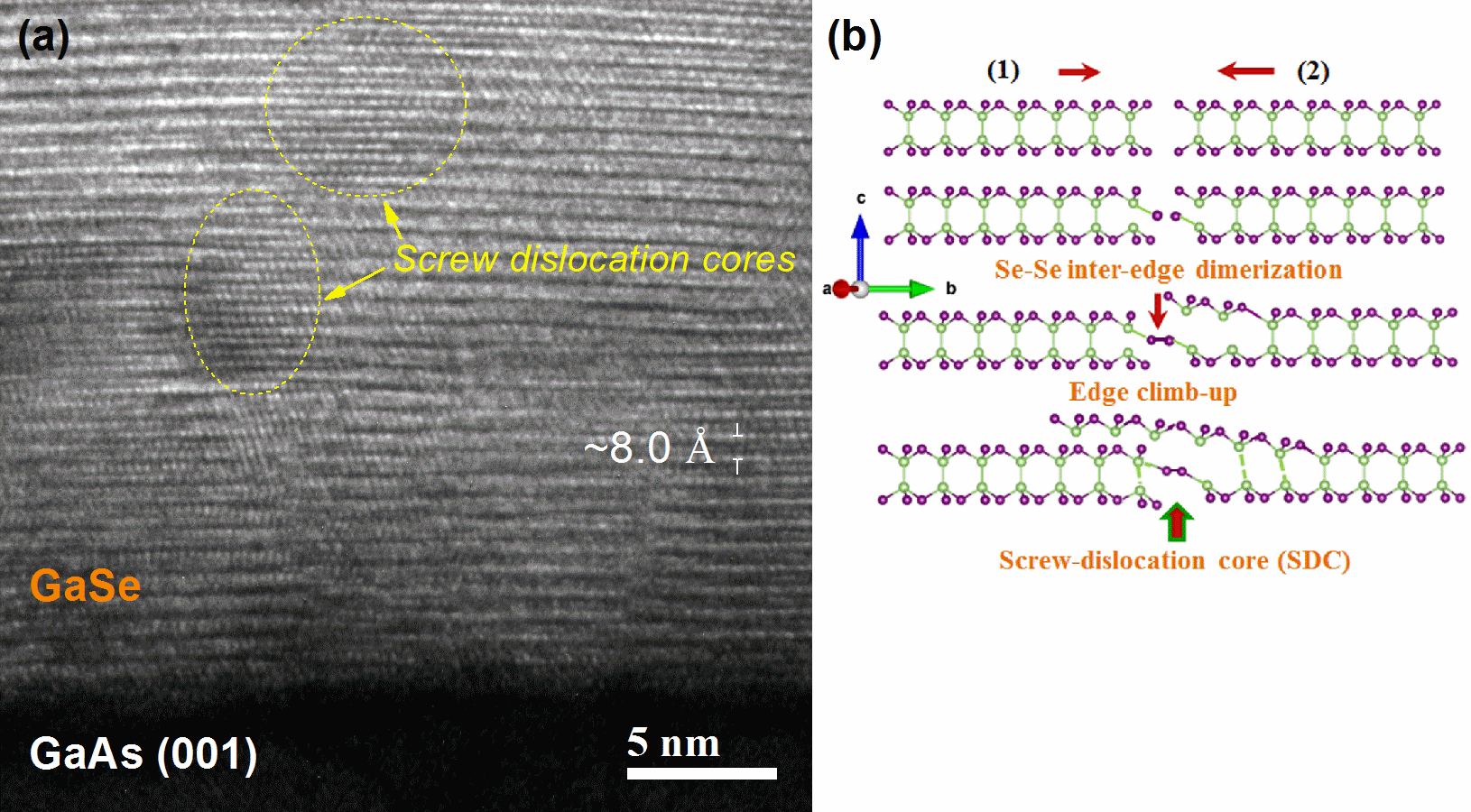


**Figure S6.** (a) Cross-sectional TEM of the SDD-GaSe film grown on GaAs (001) substrate which is observable a large number of screw-dislocation cores (reproduced from our previous work)^24^. (b) Visualization of the edge limb-up process near a screw dislocation core.
